# Comparison of Several Anthropometric Indices Related to Body Fat in Predicting Cardiorespiratory Fitness in School-Aged Children—A Single-Center Cross-Sectional Study

**DOI:** 10.3390/jcm12196226

**Published:** 2023-09-27

**Authors:** Maria Zadarko-Domaradzka, Marek Sobolewski, Emilian Zadarko

**Affiliations:** 1 Institute of Physical Culture Sciences, College of Medical Sciences, Rzeszow University, 35-959 Rzeszow, Poland; mzadarko@ur.edu.pl; 2 Department of Quantitative Methods Rzeszow, University of Technology, 35-959 Rzeszow, Poland; msobolew@prz.edu.pl

**Keywords:** body fat, RFM, TMI, waist–BMI ratio, WHtR, WHR, BMI, CRF, disease prevention

## Abstract

Body fat (BF) and cardiorespiratory fitness (CRF) are important health markers that ought to be considered in screening exams. The aim of this study was to assess the value of six indicators, i.e., tri-ponderal mass index (TMI), relative fat mass (RFM), waist–BMI ratio, waist-to-height ratio (WHtR), waist-to-hip ratio (WHR) and body mass index (BMI) in predicting CRF in school-aged children. The analysis was based on the data coming from the examination of 190 children participating in school physical education (PE) classes. Their body weight (BW) and height (BH), waist and hip circumference (WC; HC) and percentage of body fat (%BF) were measured; the CRF test was performed with the use of the 20 m shuttle run test (20 mSRT); peak heart rate (HRpeak) was measured; TMI, relative fat mass pediatric (RFMp), waist–BMI ratio, WHtR, BMI and WHR were calculated. Statistical analysis was mainly conducted using regression models. The developed regression models, with respect to the sex and age of the children, revealed RFMp as the strongest CRF indicator (*R*^2^ = 51.1%) and WHR as well as waist–BMI ratio as the weakest ones (*R*^2^ = 39.2% and *R*^2^ = 40.5%, respectively). In predicting CRF in school-aged children, RFMp turned out to be comparable to body fat percentage obtained by means of the bioimpedance analysis (BIA) (*R*^2^ = 50.3%), and as such it can be used as a simple screening measure in prophylactic exams of school children. All of these models were statistically significant (*p* < 0.001).

## 1. Introduction

Certain somatic measures: body weight (BW), waist circumference (WC), body fat (BF) and anthropometric indices: body mass index (BMI), waist-to-height ratio (WHtR) or waist–hip ratio (WHR) are considered potential predictors of chronic diseases and of an increased risk of all-cause mortality [1,2,3,4,5,6]. The results of studies from all around the world indicate that the assessment of anthropometric indices is of key importance, e.g., in early diagnosis and prevention of metabolic syndrome in children and youth [7], in identifying pediatric patients with cardiometabolic risk [8], in screening for high blood pressure in teenagers [9], in the assessment of central obesity [10], in prediction of early health risks related to central obesity [4] or in identifying cardiovascular disease (CVD) risk factors in an adult population [11].

The most widespread and routinely used index related to adiposity is BMI, which is recommended by the World Health Organization (WHO) as a surrogate marker of adiposity, bearing in mind that BMI categories for defining obesity vary by age and gender in infants, children and adolescents [12]. This index does not, however, describe distribution of fat tissue, nor does it distinguish fat-free mass from fat mass, and its limitations are a subject of discussion [13,14,15,16,17,18]. According to Gonzalez et al. (2017), BMI has a limited predictive value as far as the estimations of BF and fat-free body mass at the individual level are concerned and the authors question the use of BMI as a measure of body composition in clinical conditions [14]. Bray (2023) was also of a similar opinion, claiming that in an assessment of an individual patient, apart from BMI, other additional information is necessary, in particular that concerning the distribution of body fat, which requires additional measurements. On the other hand, he underlined that there is currently no other tool better than BMI for tracking populational changes [18].

There is a constant search for new indices and formulas related to adiposity that would allow for a better estimation of health risk measure and would be an alternative to BMI, e.g., body adiposity index (BAI), tri-ponderal mass index (TMI), relative fat mass (RFM) or waist–BMI ratio [19,20,21,22,23].

The creators of newer and newer indices imply that theirs are the indices that are more accurate in estimating body fat than BMI. According to Liu et al. (2021), the new index calculated as a multiplication of WC and BMI (waist–BMI ratio) offers “an immense potential risk marker for obesity in the clinical setting” [23]. Peterson et al. (2017) believed that TMI estimates the level of body fat better than BMI and diagnoses overweight adolescents [20]. On the other hand, the creators of RFM (a linear equation based on anthropometry) suggested that RFM is more accurate than BMI for estimations of body fat in the whole body of both sexes, both in adults [21] and children and adolescents [22]. Further studies conducted among adolescents from Brazil showed that TMI was better than RFM and BMI in predicting %BF [24]. A systematic review covering a total of 32 studies showed that TMI has a similar or a better ability to predict body fat among children and adolescents than BMI [25]. The results of Guzmán-León (2019) suggest that, compared to BMI, RFM is a better predictor of FM%—determined by each of the body composition methods [26]. Finally, Paek et al. (2019) showed that RFM has the diagnostic accuracy in detecting excessive fat tissue comparable to BMI [27].

A systematic review of the topic’s literature based on studies from 26 countries identified 25 new anthropometric measures that help to diagnose obesity and diseases related to it in adults [28].

Apart from body fat (BF), an essential variable, most strongly associated with health results, is cardiorespiratory fitness (CRF). These two variables have been considered key markers of health, which should be assessed in screening tests [29,30,31,32].

In the case of adolescents, CRF is a predictor of, among others, cardiometabolic health, mental health and academic achievement [33]. Good CRF helps to prevent chronic diseases as well as decreases the risk of premature death, and clinicians should be aware that a low level of physical activity (PA) and/or CRF may cause health damage similar to that related to obesity and cigarette smoking [34]. Assessment of CRF provides additional possibilities of advising patients in the scope of advantages of PA and improves the accuracy of health prognosis [35].

The accurate assessment of CRF and BF levels requires specialistic equipment. Hence, CRF is often estimated by means of field-based tests, e.g., the 20 m shuttle run test (20 mSRT) and predictive equations [33,36,37,38,39,40,41]; on the other hand, for BF distribution, for the estimation of %BF as well as for determining central obesity, WC measurements, anthropometric indices and estimating equations are used [10,17]. In both cases (BF ad CRF), it is much simpler, cheaper and does not require specialistic equipment or laboratory conditions.

Numerous studies conducted so far have concerned predictive potential of the so-called old indices, that is BMI, WHtR or WHR, commonly used in clinical and epidemiological studies, while the so-called novel indices still require analyzing.

That is why this study comprised, apart from the old indices, (also the) novel ones (worked out within the last 5–6 years) and their predictive potential with respect to CRF being compared. Understanding advantages and limitations of various anthropometric measures as health risk predictors is important for maintaining and promoting health as well as prophylactic measures applied from a very young age.

Non-exercise predictive models used in seemingly healthy persons have their limitations; yet, they provide a convenient CRF assessment without the need for performing maximal or submaximal exercise tests. This approach is inexpensive and does not require specialist equipment. A research review revealed [42] that from among old anthropometric indices, BMI is the most often used to predict CRF. Other variables, such as body fat percentage and waist circumference, are also used relatively frequently [43]. WHR and WHtR are also used for predicting CRF, albeit less often [44]. In the context of scientific enquiries, it seems worth drawing attention to correlations between CRF and the relatively novel anthropometric indices, i.e., RFM, TMI and waist–BMI ratio, as potential CRF predictors, which have not been presented in this capacity

The aim of this study was to assess the value of six indicators, i.e., TMI, RFMp (relative fat mass pediatric), waist–BMI ratio, WHtR, WHR and BMI in predicting CRF in school-aged children.

## 2. Materials and Methods

### 2.1. Research Procedures and Participants

The data for the analysis was obtained from a test administered to 190 participants (children between the ages of 10 and 15 inclusive), taking school physical education (PE) lessons. The test took place in 2017 (having been approved by the Bioethics Committee of the Rzeszów University—no. 1/06/2014 and with all the required consent procured). The children who did not have the necessary consent or who did but were not present when the tests were being administered were not included in the project (whose details are available in the previous publication [45]). Within the tests, anthropometric measurements and a CRF test were performed, and data on the age and sex of the tested children were collected.

### 2.2. Anthropometric Measurements and CRF Measurement

The measurements were taken by trained staff, according to the protocol, and the procedures for BW, body height (BH), %BF, WC and hip circumference (HC) measurement as well as for the assessment of the CRF are described in detail elsewhere [45]. In brief, BH and the circumferences were measured to the nearest 0.001 m with stadiometer Seca 213 (Germany, Hamburg) and Baseline Gulick anthropometric tape, respectively, while %BF was assessed with a Tanita TBF 300 (Japan, Tokyo, Tanita Corporation) employing BIA (electrical bioimpedance analysis).

The assessment of CRF was executed with the use of the 20 m shuttle run test [43] conducted in a gym and administered to 10 children at a time. The task of each participant was to perform a shuttle run covering a distance of 20 m. The distance, measured with a tape, was indicated with cones and a line. The speed of the run increased incrementally, regulated by timed sound signals (beginning at 8.5 km/h and going up by 0.5 km/h with every stage).

All the children were equipped with sports testers Polar Team2 (Polar Electro Oy, Kempele, Finland) (each child had a separate transmitter and receiver). Each child was assigned a guardian who supervised the correct performance of the test and recorded the number of completed laps as well as the obtained HR_peak_ values.

### 2.3. Statistical Analysis

Based on the data from the anthropometric measurements, the particular indices were computed as follows:

WHR = waist circumference (cm)/hip circumference (cm); (1)

BMI = weight (kg)/height ^2^ (m); (2)

WHtR = waist circumference (cm)/height (cm); (3)

TPI = body weight (kg)/body height^3^ (m); (4)

Waist–BMI ratio = waist circumference (cm)/body mass index (kg/m^2^); (5)

RFMp for girls and boys = 74 − (22 × (height/waist)) + (5 × sex *); (6)

* sex equals 0 for boys and 1 for girls.

The descriptive statistics for particular variables included arithmetic means, standard deviations (Sd) as well as maximum (Max) and minimum (Min) values. While presenting the results, the division of the tested children into 3 age groups was adopted: 10–11 years, 12–13 years, 14–15 years, which resulted from the low number of children; in the analysis of statistics (regression models, correlations), age was considered a numeric variable.

Regression analysis was used so as to search for correlations between the anthropometric indices and the CRF test results. Six models were constructed in which WHR, BMI, WHtR, TMI, waist–BMI ratio and RFMp were entered consecutively as independent variables. The results were supplemented with two additional non-exercise CRF predictive models based on the most commonly used adiposis measures, i.e., WC and %BF [43]. Sex and age were also introduced into the models. The predictive values of individual indices were compared based on the coefficient of determination (*R*^2^). By means of the stepwise regression, both the backward and the forward one, the optimal multivariable model was searched for. The values of *p* ≤ 0.05 were regarded as statistically significant. For the purpose of statistical analyses, Statistica 13.3 (TIBCO Software Inc., Palo Alto, CA, USA) was used.

## 3. Results

### 3.1. Characteristics of the Group (Anthropometric Measures and Indices)

The characteristics of the test group (N = 190, including 111 girls—mean age 12.5 years (Sd = 1.8) and 79 boys—mean age 12.4 years (Sd = 2.0)), with respect to the anthropometric measures and indices used in the analysis as well as to the CRF test results (completed laps total), can be found in Table 1.

### 3.2. Regression Models

Eight independent regression models were constructed, with the number of laps being a dependent variable, and age, sex as well as the analyzed indices BMI, WHR, WHtR, TMI, waist–BMI ratio, RFMp together with the values of %BF and WC were the independent factors (Table 2). All models were statistically significant, revealing relatively similar predictive values, with the coefficient of determination ranging between 39.2% and 51.1%. The highest results were obtained for RFMp and the lowest for WHR.

### 3.3. Multivariable Models

#### 3.3.1. Multivariable Model for the Whole Test Group

By means of multivariable regression analysis, an attempt was made to find a model that would allow for an as accurate as possible prognosis of the CRF test result, based on WHR, BMI, WHtR, TMI, waist–BMI ratio and RFMp. In addition to these variables, the model also considered sex and age, with regard to the obvious diversion of results for girls and boys and for children of different ages.

The optimal model was searched for with the use of the stepwise regression procedure, both the backwards and the forwards one. From among several alternative models, the one presented in Table 3 was chosen as the final result.

The model for the whole test group, created by means of regression analysis with reference to the factors of age and sex, shows that the number of stages in the CRF test decreases with the increase of the RFMp index—on average by 1.4 laps with the change in RFMp of 1. Taking into consideration the way RFMp is determined, the increase in this measure by 1 point translates to the decrease in the body height-to-waist circumference ratio by 1/22. Hence, e.g., of two people of the same body height, the person with a larger waist circumference shall have a higher RFMp, and, consequently, a worse fitness result in the CRF test.

**Figure 1:**
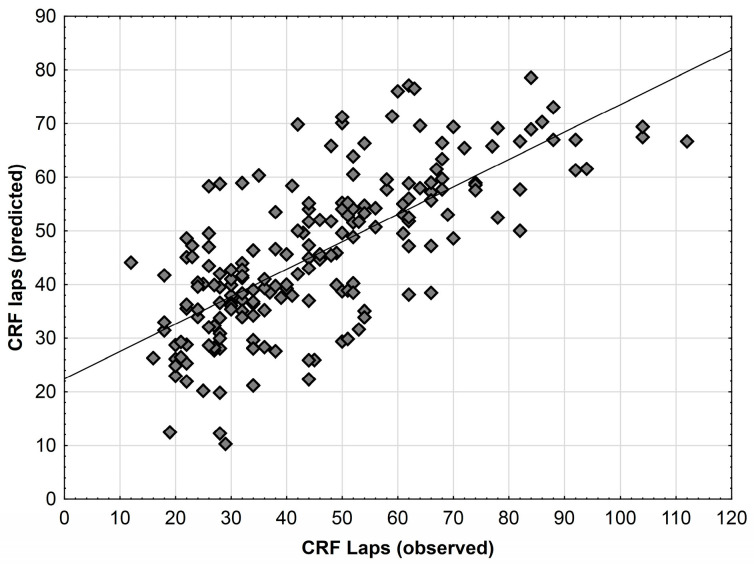
Comparison between observed and predicted values of CRF laps using models from Table 3.

The diagram shows the relationships between the CRF values (laps) observed and the values estimated based on the models from table no 3 (Figure 1).

#### 3.3.2. Multivariable Models—Separate for Girls and Boys

In the next step, models were determined, which allowed for predicting CRF separately for girls (Table 4) and boys (Table 5).

However, those results turned out to be slightly weaker than those for the combined model—the goodness of fit for each sex separately is weaker (*R*^2^ for the girls equals 32.9% and for the boys 52.9%).

For the girls, it was shown that the number of laps decreases with the decrease in WHR and the increase in waist–BMI ratio and RFMp. Judging by the value of the standardized coefficient *β*, RFMp was found to have the strongest influence on the number of laps.

Among the boys, the only risk factor for obtaining a lower number of laps is an increased RFMp, but the predictive power of the model is higher (coefficient of determination equals 52.9%).

## 4. Discussion

It is more and more often underlined that CRF assessment should be included as part of routine diagnostic procedure in all health centers [35,46]. CRF is a life parameter that should be routinely measured in clinical conditions [35]. Increased CRF is associated with lower CVD risk, regardless of BMI [15]. CRF is an important factor in determining insulin resistance in obese adolescents, regardless of their obesity category [46]. It is suggested that strategies based on early intervention and prevention aimed at CRF in adolescents may entail maintaining health parameters, decreasing the risk of obesity and cardiometabolic diseases later in life [47]. According to scientists from Estonia [48], increasing CRF in the period between the onset of puberty and adulthood is associated with a decreased risk of metabolic syndrome later in adulthood. The risk of this syndrome’s occurrence was 11.5–33.4 times higher at the age of 25 and 33 in people with persistently low CRF in comparison with people with persistently high CRF [48]. Maintaining the right level of CRF among adolescents constitutes an important strategy in the scope of public health [41].

So far, to the best of our knowledge, no studies considering RFM, TMI and waist–BMI ratio in predicting CRF have been presented. Hence, the aim of this study was to bridge this gap and decide which variable is potentially the strongest CRF predictor, also in comparison with the most popular anthropometric indices, i.e., BMI, WHR and WHtR.

Other authors have compared RFM with BMI, WC and WHR, albeit in a different context. They proved that RFM exceeds BMI in predicting general mortality and results related to liver diseases, yet they noted that RFM was not better than WHR or WC in a population-based cohort. RFM worked similarly or slightly worse than WHR and WC, both in the case of women and men [49].

The regression models for the whole test group presented in our results suggest that in predicting CRF in school-aged children, RFMp (*R*^2^ = 51.1%) is the strongest predictor in comparison with the analyzed indices such as BMI (*R*^2^ = 45.8%), WHR (*R*^2^ = 39.2%), WHtR (*R*^2^ = 50.0%), TMI (*R*^2^ = 49.1%), waist–BMI ratio (*R*^2^ = 40.5%) or WC (*R*^2^ = 47.1%) and %BF *(R*^2^ = 50.3%).

The created models, separate for the girls and for the boys, turned out to be slightly weaker than the joined model, yet in each case the model included RFMp. Among the boys, the only risk factor for obtaining a lower number of laps, which translates to a worse CRF, was a higher RFMp (*R*^2^ =52.9%). On the other hand, among the girls, it was not possible to predict the number of laps too accurately with the use of somatic indices (*R*^2^ =32.9%), yet it seems that it was valuable to establish low WHR as well as high waist–BMI ratio and RFMp as risk factors for obtaining a lower number of laps. Judging by the value of the standardized coefficient *β* = −0.83, RFMp has the strongest influence on the number of laps.

So far, studies have shown that RFM is associated with certain diseases and risk factors. A study of adult Israelis shows that RFM provides better predictability of high LDL (low-density lipoprotein), low HDL (high-density lipoprotein) and high triglycerides and metabolic syndrome than BMI [50]. Among numerous anthropometric indices of obesity, RFM displayed the strongest association with heart failure risk in adult Dutch community dwellers [51]. It is suggested that RFM may be used instead of waist circumference in order to diagnose metabolic syndrome [52]. Obesity defined through RFM predicted dyslipidemia, hypertension and adipokine imbalance disorder [53].

Studies suggest that RFM shows good correlation and a connection with BF measured by means of dual-energy X-ray absorptiometry (DXA) and BIA in young males [54]. Lokpo et al. (2023) reported that RFM had a better predictive accuracy of BIA-derived BF in females in comparison with males [55]. Compared to BMI, RFM better predicted the percentage of body fat in the whole body, measured by means of DXA among women and men [21]. Our results showed that in predicting CRF in the group of tested school-aged children, RFMp (*R*^2^ = 51.1%) was, of all the analyzed indices, the most comparable with %BF (*R*^2^ = 50.3%) measured by means of BIA.

However, the results of studies comprising youth from Brazil show that TMI was better than RFM and BMI in predicting %BF [24]. TMI may have a better predictive potential for hypertension in youth, especially among girls and older teenagers in comparison to BMI [56]. In a systematic review, comprising a total of 32 studies, the majority of the included studies suggested that TMI was similar to BMI in diagnosing metabolic syndrome, though it was suggested that TMI is a useful tool when combined with other indices (e.g., BMI and WC) [25]. According to Caiano et al. (2021), three obesity indices based on BMI, WC and RFM similarly predict first unprovoked venous thromboembolism [57].

In Poland, in compliance with an ordinance of the minister of health, all children and adolescents up to the age of 19 are entitled to free prophylactic health care, including routine screening tests and comprehensive health checks. These are not obligatory tests, though they are recommended by physicians and the Ministry of Health. Among school-aged children and youth, such comprehensive health checks occur at the ages of 9–10, 13–14, 16–17 until 19 years of age (the last grade of secondary school) and they are performed by primary healthcare doctors (GPs or pediatricians). The extent of the examination varies depending on the age, but it always comprises an interview and analyses of available medical records as well as a physical examination, which—with respect to the assessment of physical development—considers only height, body mass and BMI [58]. As indicated in the introduction, BMI should not be the only index applied in the assessment of an individual’s health. Our prior findings [45] have demonstrated that it is also worth including the measurement of WC in the examination of children and youth. This is a straightforward yet highly efficient measure of abdominal obesity whose result can also be used in calculating WHtR, commonly known and widely described in the literature. The results presented in this work again prove the usefulness of WC measurement, as it is necessary to calculate the relatively novel index RFMp, which, in our studies, turned out to be the strongest CRF predictor.

A scientific statement from the American Heart Association (2016) claims that “the addition of CRF for risk classification presents health professionals with opportunities to improve patient management and to encourage lifestyle-based strategies designed to reduce cardiovascular risk” [37].

Physical activity should be more strongly promoted in healthcare systems, given that CRF is an indicator of ordinary physical activity, in particular aerobic exercises. Clinicians should be aware that low levels of PA and/or CRF may cause harm to health [34]. The assessment of CRF and considering it as an equally important risk factor “provide additional opportunities to counsel patients on the benefits of PA” [35], as lack of physical activity and a sedentary lifestyle are serious health problems of modern societies [59]. The authors of a report on the PA of children and youth from 30 countries (including Poland) noted that the situation concerning the PA of children and youth is very concerning and unless the situation improves in this respect, a high rate of noninfectious diseases can be expected when this generation of children reaches adulthood [60].

As Ross and Myers (2023) noted, for CRF to be routinely applied in clinical practice, its measurement must be quick, simple and inexpensive [35]. By means of specialist equipment or field-based tests, the measurement of CRF in everyday medical practice is hard to achieve; hence, it is worth considering the use of the indices or equations based on anthropometric measures, such as RFMp, which are potentially the strongest CRF predictors.

In our opinion, the strength of this work lies in drawing attention to connections between CRF and the relatively new anthropometric indices in the context of their predictive potential for CRF which, so far, have not been presented. The limitations of this study lie in the limited possibility of generalizing our findings due to the low number of participants. More data are needed, from a bigger sample, to verify the results. Moreover, all the participants came from the same region of the country, so data should be collected from other regions and also from other age groups to confirm our findings.

## 5. Conclusions

In predicting CRF, RFMp turned out to be the strongest predictor in the group of school-aged children tested, comparable to the percentage of body fat obtained by means of BIA. In our assessment, this linear equation based on anthropometry can be used as a simple screening tool in prophylactic tests for school children, e.g., in children’s health checks. However, further and broader studies are needed in this respect.

## Figures and Tables

**Table 1:** The anthropometrics measures and indices of the analyzed group.

Measures and Indices	Sex	N = 190											
		10–11 Years Old				12–13 Years Old				14–15 Years Old			
		N = 68				N = 62				N = 60			
		Mean	Sd	Min	Max	Mean	Sd	Min	Max	Mean	Sd	Min	Max
BH (cm)	f	152.5	8.0	134.0	165.0	160.5	6.4	149.0	173.5	162.3	5.6	149.0	172.5
	m	148.2	7.6	131.0	168.0	163.2	10.1	148.3	180.0	172.9	7.6	160.4	190.4
BW (kg)	f	47.9	11.1	31.4	72.9	54.2	8.3	39.4	70.5	53.9	11.2	39.6	93.6
	m	46.2	12.1	24.2	67.9	53.1	14.0	34.2	84.9	60.3	11.7	41.2	88.2
HC (cm)	f	86.0	8.6	72.5	100.5	91.6	6.2	80.0	103.5	92.2	9.8	79.5	124.5
	m	83.2	10.9	65.0	102.5	86.0	9.6	71.5	108.0	90.4	7.6	79.0	104.0
WC (cm)	f	67.3	8.8	54.5	87.5	68.1	6.9	54.5	80.0	66.1	6.9	56.0	86.0
	m	70.1	11.0	55.0	90.0	69.5	7.6	58.0	89.0	71.2	7.3	61.0	90.0
BF (%)	f	23.6	7.5	8.0	37.3	26.2	6.1	9.0	40.2	21.8	7.5	7.1	37.1
	m	19.0	9.8	5.1	35.4	12.0	6.6	3.6	29.1	11.0	7.2	1.4	29.1
BMI	f	20.5	3.9	15.0	30.3	21.0	3.0	16.0	27.2	20.5	4.1	15.5	32.0
	m	20.9	4.7	14.1	29.6	19.7	3.7	15.2	29.0	20.1	3.5	14.6	27.6
WHR	f	0.78	0.06	0.68	0.96	0.74	0.05	0.63	0.84	0.72	0.03	0.66	0.78
	m	0.84	0.05	0.72	0.94	0.81	0.05	0.74	0.93	0.79	0.05	0.72	0.90
WHtR	f	0.44	0.06	0.37	0.59	0.42	0.04	0.35	0.51	0.41	0.05	0.34	0.50
	m	0.47	0.07	0.38	0.60	0.43	0.04	0.37	0.58	0.41	0.04	0.36	0.55
TMI	f	13.4	2.5	9.9	19.6	13.1	2.0	10.1	17.5	12.6	2.6	9.3	18.7
	m	14.1	3.0	10.3	20.4	12.1	2.1	9.8	18.8	11.7	2.1	8.7	16.7
Waist–BMI ratio	f	3.33	0.27	2.88	3.79	3.26	0.25	2.85	3.78	3.29	0.32	2.69	3.99
	m	3.41	0.28	2.89	3.94	3.58	0.33	3.05	4.35	3.58	0.31	3.04	4.18
RFMp	f	28.4	6.0	19.0	41.9	26.7	5.2	16.8	36.2	24.5	5.8	14.4	35.2
	m	26.5	6.6	16.3	37.5	21.9	4.8	14.1	35.9	20.1	5.1	12.4	33.8
HR_peak_ values (bpm)	f	197.4	6.9	180.0	213.0	195.7	9.5	176.0	213.0	194.5	9.2	177.0	216.0
	m	200.6	5.6	190.0	210.0	200.4	8.3	185.0	219.0	198.0	7.0	183.0	212.0
20 mSRT (laps)	f	32.8	11.4	16.0	66.0	37.8	14.0	12.0	78.0	46.7	14.8	24.0	82.0
	m	42.3	19.7	18.0	92.0	60.2	19.4	22.0	94.0	67.7	21.7	23.0	112.0

Body fat (BF); body mass index (BMI); female (f); height (BH); hip circumference (HC); 20 m shuttle run test (20 mSRT—laps); male (m); maximum values (Max); minimum values (Min); peak heart rate (HR_peak_); relative fat mass pediatric (RFMp); standard deviation (Sd); tri-ponderal mass index (TMI); waist circumference (WC); waist-to-height ratio (WHtR); waist-to-hip ratio (WHR); weight (BW).

**Table 2:** Prediction of the CRF test results (laps completed total) by means of particular somatic indicators—regression analysis results.

Models	Factors (Independent)	Regression Models—Statistics		
		*R* ^2^	*F*	*p*
1	Age, sex, WHR	39.2%	41.6	<0.0001
2	Age, sex, BMI	45.8%	54.2	<0.0001
3	Age, sex, WHtR	50.0%	64.1	<0.0001
4	Age, sex, TMI	49.1%	59.7	<0.0001
5	Age, sex, waist–BMI ratio	40.5%	42.1	<0.0001
6	Age, sex, RFMp	51.1%	65.2	<0.0001
7	Age, sex, WC	47.1%	57.2	<0.0001
8	Age, sex, %BF	50.3%	64.9	<0.0001

Coefficient of determination (*R*^2^); test statistic (*F*) and *p*-value for significance of whole model; body mass index (BMI); percentage of body fat (%BF); relative fat mass pediatric (RFMp); tri-ponderal mass index (TMI); waist circumference (WC); waist-to-height ratio (WHtR); waist-to-hip ratio (WHR).

**Table 3:** One regression model for the whole test group.

Independent Variables	Laps *R*^2^ = 51.1% *F* = 64.8 *p* < 0.0001		
	*B* (95% CI)	*p*	*β*
Intercept	45.875 (26.024; 65.726)	<0.0001	×
Sex (m vs. f)	12.286 (7.941; 16.630)	<0.0001	0.30
Age (years)	2.945 (1.776; 4.114)	<0.0001	0.27
RFMp	−1.423 (−1.787; −1.059)	<0.0001	−0.44

Male vs. female (m vs. f); coefficient of determination (*R*^2^); test statistic (*F*) and *p*-value for significance of whole model; regression coefficient with 95% CI (*B*); value for significance of each regression coefficient (*p*); value of the standardized coefficient (*β*); relative fat mass pediatric (RFMp).

**Table 4:** Regression model for girls.

Independent Variables	Laps—Girls *R*^2^ = 32.9% *F* = 13.0 *p* < 0.0001		
	*B* (95% CI)	*p*	*β*
Intercept	27.54 (−26.4; 81.47)	0.3137	×
WHR (change of 0.01)	1.11 (0.08; 2.15)	0.0349	0.42
Waist–BMI ratio (change of 0.1)	−1.61 (−3.21; −0.01)	0.0489	−0.31
RFMp (change of 1)	−2.04 (−3.09; −0.99)	0.0002	−0.83
Age (years)	2.81 (1.34; 4.27)	0.0003	0.35

Coefficient of determination (*R*^2^); test statistic (*F*) and *p*-value for significance of whole model; regression coefficient with 95% CI (*B*); value for significance of each regression coefficient (*p*); value of the standardized coefficient (*β*); relative fat mass pediatric (RFMp); waist-to-hip ratio (WHR).

**Table 5:** Regression model for boys.

Independent Variables	Laps—Boys *R*^2^ = 52.9% *F* = 42.8 *p* < 0.0001		
	*B* (95% CI)	*p*	*β*
Intercept	59.62 (24.27; 94.98)	0.0012	×
RFMp (change of 1)	−1.99 (−2.64; −1.35)	<0.0001	−0.55
Age (years)	3.39 (1.33; 5.46)	0.0016	0.29

Coefficient of determination (*R*^2^); test statistic (*F*) and *p*-value for significance of whole model; regression coefficient with 95% CI (*B*); value for significance of each regression coefficient (*p*); value of the standardized coefficient (*β*); relative fat mass pediatric (RFMp).

## Data Availability

The data of the present experimental study can be made available by the corresponding author upon reasonable request.
